# Supplementary material for: Estimating the effect of the 2005 change in BCG policy in England: a retrospective cohort study, 2000 to 2015
Source: Euro Surveill. 2019 Dec 5;24(49):1900220. doi: 10.2807/1560-7917.ES.2019.24.49.1900220 (PMC6905296; doi:10.2807/1560-7917.ES.2019.24.49.1900220)
Supplement: Supplement [file 1900220_ABBOTT_Supplement.pdf]

Online supplementary appendix: Estimating the effect of the 2005 change in BCG policy in England: a retrospective cohort study, 2000 to 2015

This supplementary material is hosted by *Eurosurveillance* as supporting information alongside the article Estimating the effect of the 2005 change in BCG policy in England: a retrospective cohort study, 2000 to 2010, on behalf of the authors, who remain responsible for the accuracy and appropriateness of the content. The same standards for ethics, copyright, attributions and permissions as for the article apply. Supplements are not edited by *Eurosurveillance* and the journal is not responsible for the maintenance of any links or email addresses provided therein

Model Definitions

Supplementary Table S1: Full definition of each model, ordered by increasing complexity.

Model	Description
Model 1	Poisson model adjusting for no fixed effects.
Model 2	Poisson model adjusting with fixed effects for the change in policy.
Model 3	Poisson model adjusting with fixed effects for the change in policy and incidence rates in the UK born.
Model 4	Poisson model adjusting with fixed effects for the change in policy and incidence rates in the non-UK born.
Model 5	Poisson model adjusting with fixed effects for the change in policy and incidence rates in the UK born and non-UK born populations.
Model 6	Poisson model adjusting with fixed effects for the change in policy and age.
Model 7	Poisson model adjusting with fixed effects for the change in policy, age, and incidence rates in the UK born.
Model 7 (Negative Binomial)	Negative binomial model adjusting with fixed effects for the change in policy, age, and incidence rates in the UK born.
Model 8	Poisson model adjusting with fixed effects for the change in policy, age, and incidence rates in the non-UK born.
Model 8 (Negative Binomial)	Negative binomial model adjusting with fixed effects for the change in policy, age, and incidence rates in the non-UK born.
Model 9	Poisson model adjusting with fixed effects for the change in policy, age, and incidence rates in the UK born and non-UK born populations.
Model 10	Poisson model with a random intercept for year of study entry, adjusting for no fixed effects.
Model 11	Poisson model with a random intercept for year of study entry, adjusting with fixed effects for the change in policy.
Model 12	Poisson model with a random intercept for year of study entry, adjusting with fixed effects for the change in policy and incidence rates in the UK born.
Model 13	Poisson model with a random intercept for year of study entry, adjusting with fixed effects for the change in policy and incidence rates in the non-UK born.
Model 14	Poisson model with a random intercept for year of study entry, adjusting with fixed effects for the change in policy and incidence rates in the UK born and non-UK born populations.
Model 15	Poisson model with a random intercept for year of study entry, adjusting with fixed effects for the change in policy and age.
Model 16	Poisson model with a random intercept for year of study entry, adjusting with fixed effects for the change in policy, age, and incidence rates in the UK born.
Model 16 (Negative Binomial)	Negative binomial model with a random intercept for year of study entry, adjusting with fixed effects for the change in policy, age, and incidence rates in the UK born.
Model 17	Poisson model with a random intercept for year of study entry, adjusting with fixed effects for the change in policy, age, and incidence rates in the non-UK born.
Model 17 (Negative Binomial)	Negative binomial model with a random intercept for year of study entry, adjusting with fixed effects for the change in policy, age, and incidence rates in the non-UK born.
Model 18	Poisson model with a random intercept for year of study entry, adjusting with fixed effects for the change in policy, age, and incidence rates in the UK born and non-UK born populations.

Imputation of UK birth status

As we were imputing a single variable we reformulated the imputation as a categorical prediction problem. This allowed us to use techniques from machine learning to improve the quality of our imputation, whilst also validating it using metrics supported by theory. We included year of notification, sex, age, Public Health England Centre (PHEC), occupation, ethnic group, Index of Multiple Deprivation (2010) categorised into five groups for England (IMD rank), and risk factor count (risk factors considered; drug use, homelessness, alcohol misuse/abuse and prison). However, we could not account for a possible missing not at random mechanism not captured by these covariates. To train the model we first split the data with complete UK birth status into a training set (80%), a calibration set (5%) and a test set (15%). We then fit a gradient boosted machine with the 10000 trees, early stopping (at a precision of $1e-5$, with 10 stopping rounds), a learning rate of 0.1, and a learn rate annealing of 0.99. Gradient boosted machines are a tree based method that can incorporate complex non-linear relationships and interactions. Much like a random forest model they work by ensembling a group of trees, but unlike a random forest model each tree is additive aiming to reduce the residual loss from previous trees. Once the model had been fit to the training set we performed platt scaling using the calibration data set. Our fitted imputation model had a Logloss of 0.28 on the test set, with an AUC of 0.93, both of which indicate a robust out of bag performance. We found that ethnic group was the most important variable for predicting UK birth status, followed by age and PHEC.

Using the fitted model we predicted the birth status for notifications where this was missing, using the F1 optimal threshold as our probability cut-off. It is common to impute missing values multiple times, to account for within- and between imputation variability. However, we considered this unnecessary for our analysis as the amount of missing data was small, our analysis considered only aggregate counts, our model metrics indicated a robust level of performance out of bag and any unaccounted for uncertainty would be outweighed by the uncertainty in our population denominator[10]. We found that cases with imputed birth status had a similar proportion of UK born to non-UK born cases as in the complete data (Supplementary Table S6).

Supplementary Table S6: Comparison of UK birth status in cases with complete or imputed records.

Status	Birth Status	Proportion of Cases (%)	Cases
Complete			106765
	UK Born	27.3	29096
	Non-UK Born	72.7	77669
Imputed			8055
	UK Born	32.7	2634
	Non-UK Born	67.3	5421

Inclusion of imputed values for UK birth status should reduce bias caused by any missing not at random mechanism captured by predictors included in the model. Graphical evaluation of UK birth status indicated that missingness has reduced over time, indicating a missing not at random mechanism. If only the complete case data then incidence rates would have reduced over the study period due to this mechanism, this may have biased our estimate of the impact of the change in policy.

Prior choice

Default weakly informative priors were used based on those provided by the brms package. For the population-level effects this was an improper flat prior over the reals. For both the standard deviations of group level effects and the group level intercepts this was a half student-t prior with 3 degrees of freedom and a scale parameter that depended on the standard deviation of the response after applying the link function.

Estimating the magnitude of the estimated impact of the change in BCG policy

We estimated the magnitude of the estimated impact from the change in BCG policy by applying the IRR estimates from the best fitting model for each cohort to the observed number of notifications from 2005 until 2015 in our study population. For the cohorts relevant to the universal school-age vaccination scheme we estimated the number of prevented cases by first aggregating cases (C_O) and then using the following equation,

$$C_P^i = C_O * (1 - I^i), \text{ Where } i = e,\ l,\ u.$$

Where C_P^i is the predicted number of cases prevented using the mean (e), 2.5% bound (l) and 97.5% bound (u) of the IRR estimate I^i. For the cohorts relevant to the targeted high-risk neonatal scheme we used a related equation, adjusting for the fact that the populations were exposed to the scheme and we therefore had to first estimate the number of cases that would have been observed had the scheme not been implemented. After simplification this results in the following equation,

$$C_P^i = C_0 \left(\frac{1}{I^i} - 1\right), \text{ Where } i = e,\ l,\ u.$$

Descriptive analysis of age-specific incidence rates

From 2000 until 2012 incidence rates in the UK born remained relatively stable but have since fallen year on year. In comparison incidence rates in the non-UK born increased from 2000 until 2005, since when they have also decreased year on year. In 14-19 year old's, who were UK born, incidence rates remained relatively stable throughout the study period, except for the period between 2006 to 2009 in which they increased year on year. This trend was not observed in the non-UK born population aged 14-19, where incidence rates reached a peak in 2003, since when they have consistently declined. In those aged 0-5, who were UK born, incidence rates also increased year on year after the change in BCG policy, until 2008 since when they have declined. This does not match with the observed trend in incidence rates in the non-UK born population, aged 0-5, in which incidence rates declined steeply between 2005 and 2006, since when they have remained relatively stable (Supplementary Figure S1; Supplementary Table S7; Supplementary Table S8).

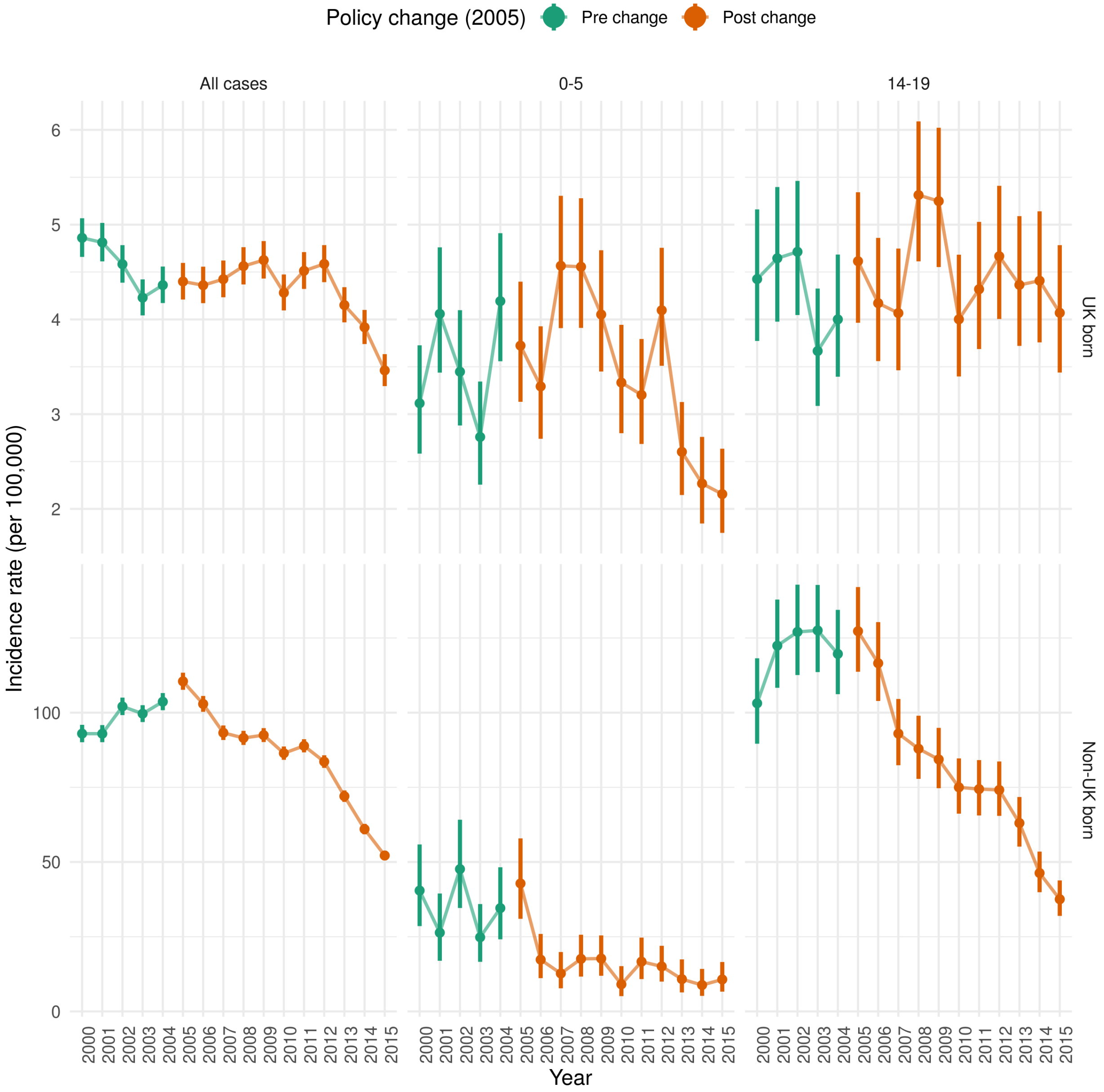

Supplementary Figure S1: Incidence rates per 100,000 for UK born population and non-UK born population, aged 0-5 and therefore directly affected by the targeted neonatal vaccination programme, and aged 14-19 and therefore directly affected by the universal school-age scheme.

Incidence estimates for all cases, those aged 0-5 and those aged 14-19

Supplementary Table S7: Incidence rates per 100,000 in the UK born for all cases, those aged 0-5, and those aged 14-19, who were directly affected by the change in vaccination policy in 2005

Year eligible for vaccination	Age group		
	All cases*	0-5*	14-19*
2000	4.86 (4.66, 5.07)	3.12 (2.58, 3.73)	4.43 (3.77, 5.16)
2001	4.81 (4.61, 5.02)	4.06 (3.44, 4.76)	4.65 (3.98, 5.40)
2002	4.58 (4.39, 4.78)	3.45 (2.88, 4.10)	4.71 (4.05, 5.46)
2003	4.23 (4.04, 4.42)	2.76 (2.26, 3.34)	3.67 (3.09, 4.33)
2004	4.36 (4.17, 4.56)	4.19 (3.56, 4.91)	4.00 (3.40, 4.68)
2005	4.40 (4.21, 4.60)	3.72 (3.13, 4.40)	4.61 (3.96, 5.34)
2006	4.36 (4.17, 4.56)	3.29 (2.74, 3.93)	4.17 (3.56, 4.86)
2007	4.42 (4.23, 4.62)	4.57 (3.91, 5.30)	4.07 (3.46, 4.75)
2008	4.56 (4.37, 4.76)	4.56 (3.91, 5.28)	5.31 (4.61, 6.09)
2009	4.63 (4.43, 4.83)	4.05 (3.45, 4.73)	5.25 (4.55, 6.02)
2010	4.28 (4.09, 4.47)	3.33 (2.80, 3.94)	4.00 (3.40, 4.68)
2011	4.51 (4.32, 4.71)	3.20 (2.69, 3.79)	4.32 (3.69, 5.03)
2012	4.59 (4.39, 4.78)	4.10 (3.51, 4.76)	4.67 (4.00, 5.41)
2013	4.15 (3.97, 4.34)	2.60 (2.15, 3.13)	4.36 (3.72, 5.09)
2014	3.92 (3.74, 4.10)	2.27 (1.85, 2.76)	4.41 (3.76, 5.14)
2015	3.46 (3.30, 3.63)	2.16 (1.75, 2.64)	4.07 (3.44, 4.78)

* Incidence rate per 100,000, with 95% confidence intervals

Supplementary Table S8: Incidence rates per 100,000 in the non-UK born for all cases, those aged 0-5, and those aged 14-19, who would have been directly affected by the change in vaccination policy in 2005 had they been UK born

Year eligible for vaccination	Age group		
	All cases*	0-5*	14-19*
2000	92.98 (90.10, 95.92)	40.45 (28.56, 55.88)	103.14 (89.60, 118.19)
2001	92.95 (90.12, 95.84)	26.36 (16.95, 39.47)	122.40 (108.32, 137.85)
2002	102.07 (99.18, 105.03)	47.63 (34.62, 64.16)	127.03 (112.59, 142.83)
2003	99.65 (96.85, 102.50)	24.81 (16.59, 35.94)	127.53 (113.57, 142.75)
2004	103.66 (100.82, 106.56)	34.58 (24.13, 48.25)	119.66 (106.18, 134.41)
2005	110.48 (107.64, 113.37)	42.83 (30.99, 57.91)	127.26 (113.69, 142.04)
2006	102.91 (100.28, 105.59)	17.32 (11.13, 25.93)	116.54 (103.91, 130.31)
2007	93.26 (90.85, 95.71)	12.69 (7.74, 19.87)	92.99 (82.40, 104.58)
2008	91.52 (89.19, 93.90)	17.59 (11.66, 25.67)	87.92 (77.84, 98.97)
2009	92.47 (90.17, 94.82)	17.69 (11.92, 25.44)	84.34 (74.71, 94.90)
2010	86.41 (84.21, 88.67)	9.07 (5.11, 15.16)	75.00 (66.19, 84.68)
2011	88.88 (86.70, 91.10)	16.65 (10.82, 24.70)	74.41 (65.59, 84.12)
2012	83.60 (81.51, 85.73)	15.05 (9.97, 21.96)	74.12 (65.45, 83.65)
2013	72.03 (70.13, 73.97)	10.80 (6.36, 17.41)	63.04 (55.16, 71.77)
2014	61.01 (59.29, 62.78)	8.82 (5.19, 14.22)	46.31 (39.90, 53.49)
2015	52.18 (50.62, 53.77)	10.69 (6.62, 16.54)	37.55 (31.97, 43.83)

* Incidence rate per 100,000, with 95% confidence intervals

Direct effects of the change in policy on the UK born cohorts - results from all models

Supplementary Table S2: Comparison of models fitted to incidence rates for the UK born population that were relevant to the universal vaccination programme of those at school-age (14). Models are ordered by the goodness of fit as assessed by LOOIC, the degrees of freedom are used as a tiebreaker.

Model	IRR (CrI 95%)*	Variable					DoF**	LPD†	LOOIC (se)††
		Policy Change	Age	UK born rates	Non-UK born rates	Year of study entry			
Model 7 (Negative Binomial)	1.08 (0.97, 1.19)	Yes	Yes	Yes	No	No	9	-211	439 (10)
Model 7	1.08 (1.00, 1.17)	Yes	Yes	Yes	No	No	8	-211	443 (14)
Model 9	1.12 (1.01, 1.25)	Yes	Yes	Yes	Yes	No	9	-210	445 (14)
Model 16	1.08 (0.97, 1.21)	Yes	Yes	Yes	No	Yes	20	-207	445 (14)
Model 18	1.12 (0.97, 1.28)	Yes	Yes	Yes	Yes	Yes	21	-207	447 (15)
Model 8	1.16 (1.04, 1.29)	Yes	Yes	No	Yes	No	8	-213	449 (17)
Model 6	1.06 (0.98, 1.15)	Yes	Yes	No	No	No	7	-215	452 (17)
Model 17	1.15 (1.00, 1.32)	Yes	Yes	No	Yes	Yes	20	-209	452 (17)
Model 15	1.06 (0.94, 1.20)	Yes	Yes	No	No	Yes	19	-209	453 (17)
Model 1	0.00 (0.00, 0.00)	No	No	No	No	No	1	-254	513 (26)
Model 2	1.06 (0.98, 1.14)	Yes	No	No	No	No	2	-252	515 (25)
Model 4	1.00 (0.90, 1.10)	Yes	No	No	Yes	No	3	-251	516 (25)
Model 3	1.06 (0.98, 1.15)	Yes	No	Yes	No	No	3	-252	518 (26)
Model 5	0.98 (0.89, 1.09)	Yes	No	Yes	Yes	No	4	-249	518 (24)
Model 13	0.94 (0.78, 1.12)	Yes	No	No	Yes	Yes	15	-237	518 (27)
Model 10	0.00 (0.00, 0.00)	No	No	No	No	Yes	13	-244	521 (28)
Model 11	1.06 (0.94, 1.20)	Yes	No	No	No	Yes	14	-244	522 (28)
Model 14	0.93 (0.78, 1.11)	Yes	No	Yes	Yes	Yes	16	-236	522 (27)
Model 12	1.06 (0.93, 1.20)	Yes	No	Yes	No	Yes	15	-243	526 (28)

* Incidence Rate Ratio, with 95% credible intervals,
** Degrees of Freedom,
† Computed log pointwise predictive density,
†† Leave one out information criterion with standard error

Supplementary Table S4: Comparison of models fitted to incidence rates for the UK born population that were elgible to the targeted vaccination programme of neonates. Models are ordered by the goodness of fit as assessed by LOOIC, the degrees of freedom are used as a tiebreaker.

Model	IRR (CrI 95%)*	Variable					DoF**	LPD†	LOOIC (se)††
		Policy Change	Age	UK born rates	Non-UK born rates	Year of study entry			
Model 16	0.96 (0.82, 1.14)	Yes	Yes	Yes	No	Yes	20	-192	415 (12)
Model 16 (Negative Binomial)	0.96 (0.82, 1.13)	Yes	Yes	Yes	No	Yes	21	-196	415 (10)
Model 16 (Negative Binomial)	0.96 (0.82, 1.13)	Yes	Yes	Yes	No	Yes	21	-196	415 (10)
Model 18	0.99 (0.82, 1.18)	Yes	Yes	Yes	Yes	Yes	21	-192	417 (13)
Model 7	0.96 (0.88, 1.05)	Yes	Yes	Yes	No	No	8	-200	420 (15)
Model 9	1.00 (0.89, 1.12)	Yes	Yes	Yes	Yes	No	9	-200	422 (15)
Model 8	1.02 (0.91, 1.15)	Yes	Yes	No	Yes	No	8	-203	427 (16)
Model 6	0.95 (0.87, 1.03)	Yes	Yes	No	No	No	7	-204	428 (16)
Model 15	0.95 (0.83, 1.09)	Yes	Yes	No	No	Yes	19	-198	428 (14)
Model 17	1.02 (0.87, 1.20)	Yes	Yes	No	Yes	Yes	20	-198	429 (14)
Model 14	1.10 (0.92, 1.33)	Yes	No	Yes	Yes	Yes	16	-206	442 (16)
Model 5	1.08 (0.97, 1.21)	Yes	No	Yes	Yes	No	4	-216	445 (18)
Model 12	0.98 (0.83, 1.15)	Yes	No	Yes	No	Yes	15	-209	448 (17)
Model 4	1.12 (1.00, 1.24)	Yes	No	No	Yes	No	3	-219	449 (18)
Model 3	0.97 (0.89, 1.06)	Yes	No	Yes	No	No	3	-219	450 (19)
Model 13	1.14 (0.97, 1.35)	Yes	No	No	Yes	Yes	15	-211	452 (16)
Model 1	0.00 (0.00, 0.00)	No	No	No	No	No	1	-229	462 (21)
Model 2	0.95 (0.87, 1.03)	Yes	No	No	No	No	2	-228	463 (20)
Model 10	0.00 (0.00, 0.00)	No	No	No	No	Yes	13	-220	466 (19)
Model 11	0.95 (0.83, 1.09)	Yes	No	No	No	Yes	14	-219	467 (19)

* Incidence Rate Ratio, with 95% credible intervals,
** Degrees of Freedom,
† Computed log pointwise predictive density,
†† Leave one out information criterion with standard error

Direct effects of the change in policy on the non-UK born cohorts - results from all models

Supplementary Table S3: Comparison of models fitted to incidence rates for the non-UK born population that were eligible to the universal vaccination programme of those at school-age (14). Models are ordered by the goodness of fit as assessed by LOOIC, the degrees of freedom are used as a tiebreaker.

Model	IRR (CrI 95%)*	Variable					DoF**	LPD†	LOOIC (se)††
		Policy Change	Age	UK born rates	Non-UK born rates	Year of study entry			
Model 17 (Negative Binomial)	0.74 (0.61, 0.88)	Yes	Yes	No	Yes	Yes	21	-228	483 (10)
Model 17	0.74 (0.62, 0.87)	Yes	Yes	No	Yes	Yes	20	-223	492 (16)
Model 18	0.73 (0.61, 0.87)	Yes	Yes	Yes	Yes	Yes	21	-222	493 (16)
Model 15	0.64 (0.53, 0.78)	Yes	Yes	No	No	Yes	19	-224	496 (18)
Model 16	0.65 (0.54, 0.78)	Yes	Yes	Yes	No	Yes	20	-223	496 (17)
Model 8	0.79 (0.73, 0.86)	Yes	Yes	No	Yes	No	8	-239	507 (20)
Model 9	0.79 (0.72, 0.86)	Yes	Yes	Yes	Yes	No	9	-238	511 (20)
Model 11	0.64 (0.52, 0.78)	Yes	No	No	No	Yes	14	-241	522 (22)
Model 10	0.00 (0.00, 0.00)	No	No	No	No	Yes	13	-241	523 (22)
Model 12	0.64 (0.53, 0.79)	Yes	No	Yes	No	Yes	15	-241	525 (22)
Model 13	0.64 (0.52, 0.79)	Yes	No	No	Yes	Yes	15	-241	526 (23)
Model 14	0.64 (0.52, 0.79)	Yes	No	Yes	Yes	Yes	16	-241	530 (23)
Model 7	0.66 (0.62, 0.70)	Yes	Yes	Yes	No	No	8	-248	532 (23)
Model 6	0.65 (0.61, 0.69)	Yes	Yes	No	No	No	7	-253	539 (27)
Model 4	0.70 (0.65, 0.76)	Yes	No	No	Yes	No	3	-270	556 (31)
Model 5	0.70 (0.64, 0.76)	Yes	No	Yes	Yes	No	4	-270	559 (31)
Model 2	0.65 (0.61, 0.69)	Yes	No	No	No	No	2	-275	561 (33)
Model 3	0.65 (0.61, 0.69)	Yes	No	Yes	No	No	3	-273	561 (32)
Model 1	0.00 (0.00, 0.00)	No	No	No	No	No	1	-341	692 (51)

* Incidence Rate Ratio, with 95% credible intervals,

** Degrees of Freedom,

† Computed log pointwise predictive density,

†† Leave one out information criterion with standard error

Supplementary Table S5: Comparison of models fitted to incidence rates for the non-UK born population that were revelant to the targeted vaccination programme of neonates. Models are ordered by the goodness of fit as assessed by LOOIC, the degrees of freedom are used as a tiebreaker.

Model	IRR (CrI 95%)*	Variable					DoF**	LPD†	LOOIC (se)††
		Policy Change	Age	UK born rates	Non-UK born rates	Year of study entry			
Model 8 (Negative Binomial)	0.62 (0.44, 0.88)	Yes	Yes	No	Yes	No	9	-138	293 (15)
Model 8	0.64 (0.47, 0.86)	Yes	Yes	No	Yes	No	8	-137	295 (18)
Model 9	0.62 (0.45, 0.85)	Yes	Yes	Yes	Yes	No	9	-137	297 (18)
Model 6	0.47 (0.38, 0.58)	Yes	Yes	No	No	No	7	-139	298 (19)
Model 7	0.48 (0.39, 0.60)	Yes	Yes	Yes	No	No	8	-139	298 (19)
Model 17	0.63 (0.44, 0.89)	Yes	Yes	No	Yes	Yes	20	-135	298 (18)
Model 18	0.61 (0.42, 0.87)	Yes	Yes	Yes	Yes	Yes	21	-135	300 (18)
Model 15	0.47 (0.35, 0.62)	Yes	Yes	No	No	Yes	19	-136	301 (20)
Model 16	0.48 (0.36, 0.63)	Yes	Yes	Yes	No	Yes	20	-136	301 (19)
Model 4	0.82 (0.61, 1.10)	Yes	No	No	Yes	No	3	-147	304 (17)
Model 5	0.78 (0.58, 1.06)	Yes	No	Yes	Yes	No	4	-147	306 (18)
Model 13	0.83 (0.59, 1.16)	Yes	No	No	Yes	Yes	15	-145	308 (18)
Model 14	0.78 (0.55, 1.12)	Yes	No	Yes	Yes	Yes	16	-144	310 (19)
Model 3	0.52 (0.42, 0.64)	Yes	No	Yes	No	No	3	-152	314 (22)
Model 12	0.51 (0.38, 0.69)	Yes	No	Yes	No	Yes	15	-148	317 (23)
Model 2	0.49 (0.40, 0.61)	Yes	No	No	No	No	2	-156	319 (22)
Model 11	0.49 (0.37, 0.65)	Yes	No	No	No	Yes	14	-152	322 (23)
Model 10	0.00 (0.00, 0.00)	No	No	No	No	Yes	13	-150	330 (25)
Model 1	0.00 (0.00, 0.00)	No	No	No	No	No	1	-171	346 (27)

* Incidence Rate Ratio, with 95% credible intervals,
** Degrees of Freedom,
† Computed log pointwise predictive density,
†† Leave one out information criterion with standard error
